# A direct quantification method for measuring plasma MicroRNAs identified potential biomarkers for detecting metastatic breast cancer

**DOI:** 10.18632/oncotarget.7990

**Published:** 2016-03-08

**Authors:** Qian Zhao, Shengqiong Deng, Guangxue Wang, Cuicui Liu, Lingyu Meng, Shanshan Qiao, Lei Shen, Yue Zhang, Jinhui Lü, Wenshu Li, Yuzhen Zhang, Min Wang, Richard G. Pestell, Chunli Liang, Zuoren Yu

**Affiliations:** ^1^ Research Center for Translational Medicine, Translational Medical Center for Stem Cell Therapy, Shanghai East Hospital, Tongji University School of Medicine, Shanghai, China; ^2^ Shanghai East Hospital, Dalian Medical University, Dalian, China; ^3^ School of Basic Medical Sciences, Wenzhou Medical University, Wenzhou, China; ^4^ Department of Cancer Biology, Sidney Kimmel Cancer Center, Thomas Jefferson University, Philadelphia, USA

**Keywords:** circulating miRNA, direct quantification, biomarker, metastatic breast cancer, miR-106a

## Abstract

Circulating miRNAs are protected from ribonuclease degradation by assembly into microvesicles and exosomes. Releasing miRNAs completely from these particles is the key step to quantify the circulating miRNAs. Currently purified RNA-based quantitative analysis is widely used while it is time and cost consuming with high risk for those circulating miRNAs with low abundance due to partial loss of RNA during the steps of total RNA extraction and small RNA enrichment. Herein, we optimized a simple, effective and time-saving method to directly measure plasma miRNAs without RNA isolation. It is based on complete miRNA release from the protein complexes, followed by miRNA-specific reverse transcription and quantitative real-time PCR amplification. By comparison to the RNA-based approach, the direct quantification method showed more efficiency for circulating miRNA analysis, higher accuracy and specificity. By application of the direct quantification method to clinical samples combined with the RNA-based miRNA screening analysis, upregulation of miR-106a in blood was validated in metastatic breast cancer patients, indicating miR-106a are a potential biomarker for metastatic breast cancer.

## INTRODUCTION

The Human Genome Project has demonstrated that more than 93% of the genome DNA can be transcribed to RNA while less than 2% of transcripts encode functional proteins [1], indicating most of the transcripts are non-coding RNAs (ncRNA). Those ncRNAs with length < 200 nt are called small non-coding RNAs and include transfer RNA (tRNA), ribosomal RNA (rRNA), small nuclear RNA (snoRNA), microRNA (miRNA), small interference RNA (siRNA) and piwi-interacting RNA (piRNA).

miRNAs are a class of multifunctional singled-stranded small molecules that regulate the stability or translational efficiency of targeted messenger RNAs. Mature miRNAs have length ranging from 18 to 24 nt. More than 2,000 miRNA sequences have been identified or predicted from human-origin cells. miRNAs can be assembled into ribonucleic protein complexes known as RNA-induced silencing complex (RISC) that lead to base-pairing interactions between a miRNA and the binding site of its target mRNAs mostly within the 3′ untranslated region (3′UTR). miRNAs regulate diverse biological processes including cell fate determination, cell cycle progression, stem cell self-renewal, cancer initiation, cancer cell proliferation and cancer cell metastasis [2–8].

miRNAs are present in body fluids including blood, plasma, serum, saliva, urine and milk [9–12]. The extracellular miRNAs circulating in the blood of both healthy and diseased people, are referred to circulating miRNAs. Most of the circulating miRNAs are protected from ribonuclease degradation by cofractionating with proteins or assembling in lipid or lipoprotein complexes, such as microvesicles and/or exosomes [13, 14]. Therefore, circulating miRNAs are highly stable in blood. Although the understanding of how miRNAs are selectively released from cells and how the circulating miRNAs are related to disease remains largely unclear, circulating miRNAs may serve as novel diagnostic and prognostic biomarkers for human diseases including cancer [15]. Emerging evidence suggests secreted miRNAs function as heterotypic signals to regulate the breast cancer phenotype [16] and circulating miRNAs may serve as heterotypic signaling molecules for intercellular communication by functioning in recipient cells [17].

Breast cancer is the most common non-dermatological cancer in women. Although the 5-year survival has reached around 98% in localized breast cancer [18], metastatic breast cancer, which occurs in 20%–30% of breast cancer patients, remains incurable partly due to the lack of a reliable biomarker for early diagnosis before metastasis occurs [19]. miRNAs regulate breast cancer initiation and progression functioning as either tumor suppressors or oncogenes [6, 20]. Altered expression of miRNAs or mutation of miRNA genes have been described in human breast cancer. As early as in 2005, Lorio et al identified 29 miRNAs with aberrant expression in human breast cancer by analysis on 76 breast tumor samples and 14 human breast cell lines [21]. Zhang and his colleagues analyzed 283 human miRNA genes in 55 human breast primary tumors and 18 human breast cancer cell lines, demonstrating a high frequency (~72.8%) of gene copy number abnormality in miRNA-containing regions in human breast cancer [22]. A growing body of evidence implicates miRNAs in regulating breast cancer metastasis as well as epithelial to mesenchymal transition (EMT).

Although the function of circulating miRNAs in breast cancer is not well understood, emerging evidence suggests circulating miRNA may be important in diagnosis and prognosis of breast cancer. Circulating miRNA in blood of breast cancer patients demonstrate increased levels of miR-195 and let-7a compared to control subjects [23]. A recent report proposed miR-181 a-5p as a diagnostic breast cancer biomarker because of a significant reduction of circulating miR-181a-5p level in breast cancer patients' serum [24]. However, the circulating miRNAs in metastatic breast cancer remains to be determined.

Currently methods for circulating miRNA analysis are mainly based on RNA purification and small RNA enrichment. It is unavoidable to have part of the circulating miRNAs lost due to the incomplete denaturation of protein and/or incomplete precipitation of RNA, which may lead to failure to detect low abundance miRNAs. Yoon et al reported an assay to detect mRNA and miRNA directly from cell lysates (cell to Ct) without RNA isolation [25]. Sota et al reported a direct serum assay for miRNA analysis in cancer patient [26]. Both reports lacked evaluation of the specificity, accuracy and efficiency of the direct approach, which limits the reliability and applicability. In the present study we optimized a simple, effective and time-saving method to directly measure the plasma miRNA without RNA purification. The method is based on a complete denaturation of miRNA-containing microvesicles and/or exosomes, miRNA release from those complexes, followed by miRNA-specific reverse transcription and quantitative real-time PCR amplification (QRT-PCR). RNA isolation and purification is not required in this assay. It greatly shortens the time and decreases the cost for the circulating miRNAs measurement compared to the traditional RNA-based assays. More importantly, the direct quantification method showed more efficiency for measuring circulating miRNAs compared to the RNA-based approaches. By applying the direct quantification method, we validated changes in circulating miR-106a levels in metastatic breast cancer, and proved evidence for the potential as a biomarker specifically for metastatic human breast cancer.

## RESULTS

### Direct quantification of circulating miRNAs from plasma without RNA purification

**Figure 1:**
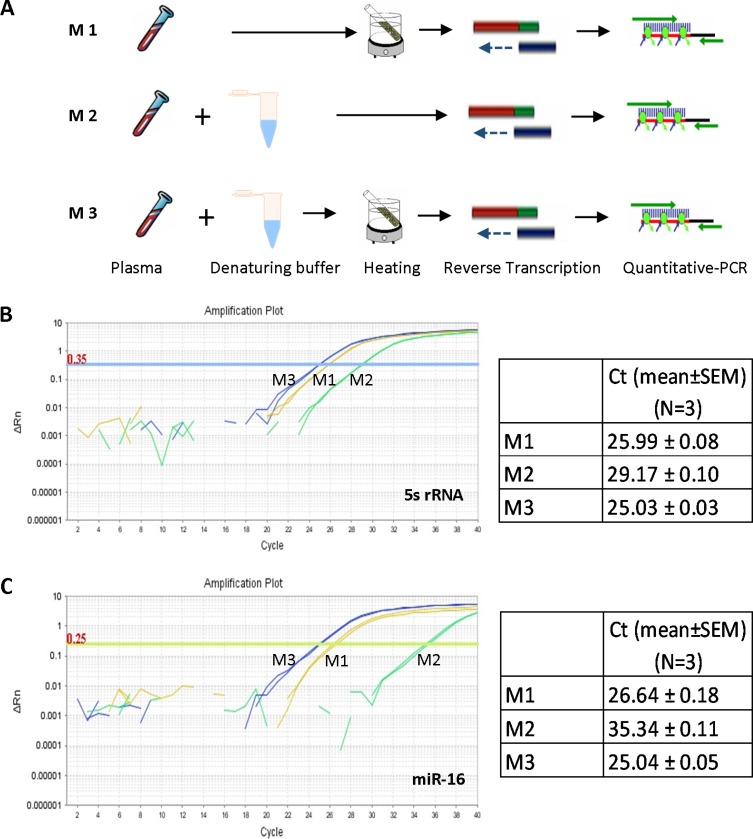
Direct quantification of circulating miRNAs using plasma (**A**) Schematic representation of the three methods (M1, M2 and M3) directly analyzing circulating miRNAs from plasma. (**B**) QRT-PCR analysis of small RNA 5s rRNA from aliquots of same plasma sample using methods M1, M2 and M3, respectively. (**C**) QRT-PCR analysis of miR-16 from aliquots of same plasma sample using methods M1, M2 and M3, respectively.

In order to determine how circulating miRNAs can be released efficiently from microvesicles and exosomes in blood and whether the released miRNAs can be directly amplified for quantitative analysis, different methods for denaturing protein, including chemical detergents and heating treatment, were compared. As shown in Figure 1A, aliquots of 5 μl plasma were treated by M1 (75°C for 5 min alone), M2 (treated with same volume of denaturing buffer containing Tween20, Tris and EDTA) or M3 (denaturing buffer reaction followed by heat treatment), respectively. The released circulating miRNAs were immediately reverse-transcribed to cDNA without further RNA purification. Using the cDNA as template, two of the most abundant small RNAs in plasma, 5s rRNA and miR-16, were amplified by QRT-PCR reaction (Figure 1B and 1C). For both 5s rRNA (Figure 1B) and miR-16 (Figure 1C), M3 showed the highest efficiency for gene amplification, M1 showed less efficiency (M1 vs M3, ΔCt ~1 cycle) while M2 showed the lowest efficiency (M2 vs M3, ΔCt ~10 cycles). The results demonstrated the feasibility of circulating miRNAs direct amplification using plasma without RNA purification, and indicated that approach M3 has the highest efficiency to release miRNAs from protein complexes for further analysis. SDS, which is used the most frequently for protein denaturation, was tested to release circulating miRNAs from protein complexes, followed by a miRNA direct quantification analysis. As shown in Supplementary Figure S1, none of the three tested small RNAs were amplified after SDS treatment, indicating the existence of SDS in the reaction system may have severe inhibition to miRNA amplification.

### The accuracy of the plasma-based direct quantification of circulating miRNAs

**Figure 2:**
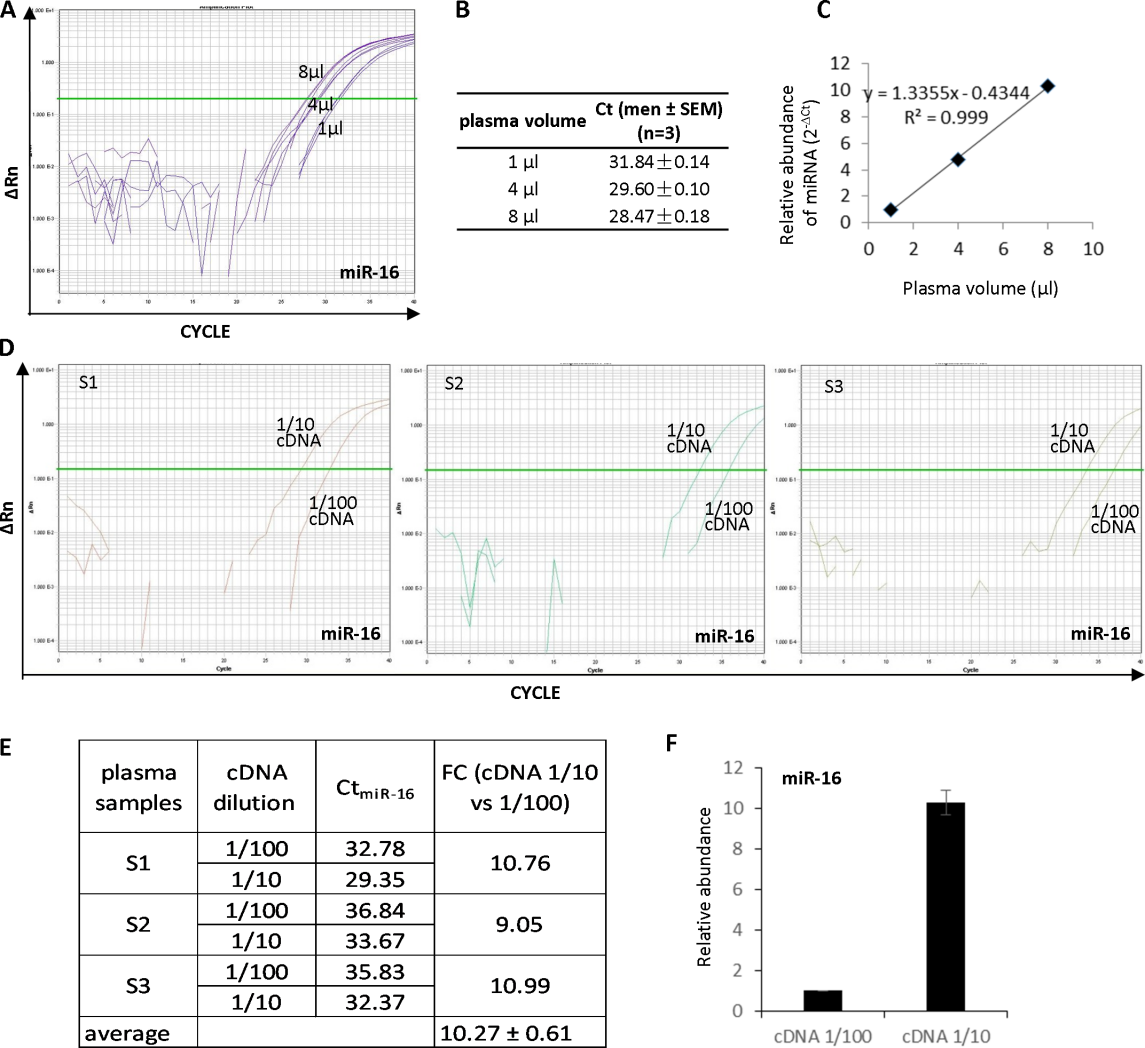
High accuracy of the plasma-based direct quantification of circulating miRNAs (**A**) Quantitative analysis of miR-16 from 1, 4 and 8 μl of plasma sample using methods M3. (**B**) Ct values in A analysis. (**C**) The miR-16 abundance showed a linear positive correlation with the plasma volume with R^2^ = 0.999. (**D**) Quantitative analysis of miR-16 from three different plasma samples (S1, S2 and S3) with 1:10 and 1:100 cDNA dilutions. (**E**) Actual Ct values and fold changes of miR-16 abundance in the three samples at cDNA dilution 1:10 vs 1:100. (**F**) miR-16 abundance showed 10.27 times more in 1:10 than 1:100 diluted cDNAs, which is consistent with cDNA concentration.

In order to evaluate the accuracy of the plasma-based method for circulating miRNA analysis, different volumes of plasma (1 μl, 4 μl, and 8 μl) from the same donor were treated in parallel using method M3, followed by reverse transcription and QRT-PCR amplification of miR-16 (Figure 2A–2C). 1 μl, 4 μl, and 8 μl of the plasma yielded Ct values around 31.8, 29.6 and 28.5 cycles, respectively (Figure 2A and 2B). The Log_2_ ΔCt had a perfect correlation with the plasma volume (Figure 2C) indicating the miRNA quantification result reflects the real abundance of miR-16 in the reaction system.

In addition, direct quantification of circulating miRNAs was performed in multiple plasma samples with different dilutions of cDNA to validate the accuracy of the method. Three plasma samples from randomly selected three donors were tested for miR-16. cDNAs from plasma specimens S1, S2 and S3 were diluted by 1:10 and 1:100, respectively (Figure 2D). Circulating miR-16 was analyzed in all diluted cDNAs. The results showed ~3.3 ΔCt between 1:10 and 1:100 diluted cDNAs in all the three tested samples (Figure 2D and 2E), representing the average fold change 10.27 ± 0.61 of the miR-16 abundance in 1:10 compared with 1:100 diluted cDNAs (Figure 2F). In addition to miR-16, miR-92a and let-7b were also measured in S1 cDNA diluted by 1:10 and 1:100. Similar to miR-16, both miR-92a (Supplementary Figure S2) and let-7b (Supplementary Figure S3) showed ΔCt around 3.3–3.5 cycles which is consistent with the 10-fold difference in cDNA concentration.

### The specificity of the plasma-based direct quantification of circulating miRNAs

**Figure 3:**
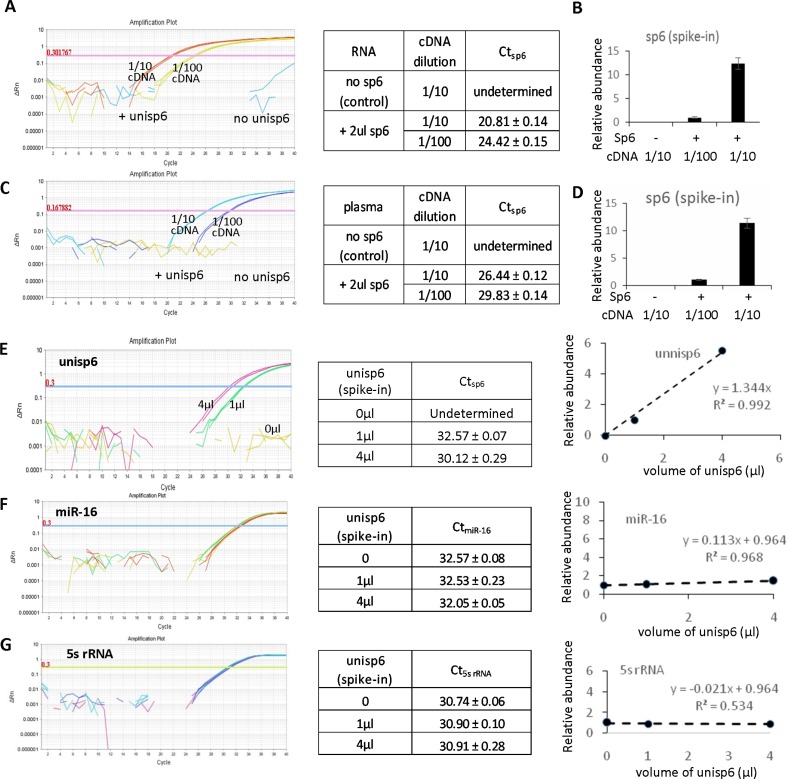
High specificity of the plasma-based direct quantification of circulating miRNAs (**A**) QRT-PCR analysis of an exogenous small RNA unisp6 which was added into purified RNA with 1:10 and 1:100 dilutions to cDNA. (**B**) Quantitative analysis of unisp6 abundance in A. (**C**) QRT-PCR analysis of exogenous small RNA unisp6 which was added into plasma with 1:10 and 1:100 dilutions to cDNA. (**D**) Quantitative analysis of unisp6 abundance in C. (**E**) Quantitative analysis of exogenous small RNA unisp6 which was added into plasma with volume 0, 1 and 4 μl. (**F**) Quantitative analysis of miR-16 in all cDNAs of E. (**G**) Quantitative analysis of 5s rRNA in all cDNAs of E.

In order to determine the specificity of the direct quantification method for circulating miRNA analysis, an exogenous small RNA named unisp6 was added into either purified RNA or the original plasma for further analysis. Firstly it was tested in the RNA sample to ensure the exogenous RNA is able to be amplified well. 2 μl of unisp6 (1 μl equals to 10^8^ copies) was added to a RNA sample followed by a miRNA reverse transcription reaction. The cDNA was diluted with 1:10 and 1:100 followed by a PCR amplification. Accordingly, the results showed Ct values 20.8 ± 0.14 and 24.4 ± 0.15 corresponding to the two diluted cDNAs (Figure 3A), while the control RNA (without adding unisp6) only had a background signal. Data analysis indicated the fold change of unisp6 abundance is consistent with the cDNA concentration (Figure 3B). Similarly, 2 μl of unisp6 was added directly to a plasma sample for a small RNA quantitative analysis using the method M3. Unisp6 was undetectable in control sample, while it had Ct values of 26.44 ± 0.12 and 29.83 ± 0.14 in 1:10 and 1:100 diluted cDNAs, respectively (Figure 3C). The ~ 10-fold change of unisp6 abundance is consistent with the cDNA concentration (Figure 3D).

In addition, different amount of unisp6 (0, 1, and 4 μl) were added into aliquots of plasma samples, followed by protein denaturation, reverse transcription and PCR amplification. As expected, unisp6 was undetectable in the reaction with 0 μl unisp6. The expression levels of unisp6 correlated with the amount of unisp6 added into the plasma (Figure 3E). As a form of control, the abundance of two endogenous small RNAs, miR-16 and 5s rRNA, did not show change between these samples (Figure 3F and 3G). Another set of experiments with 0, 2 and 4 μl of unisp6 added into a plasma sample further confirmed the specificity and accuracy of this direct quantitative method for circulating miRNA analysis (Supplementary Figure S4, S5 and S6).

The results also reflected the sensitivity of the method. As indicated in Supplementary Figure S5, 2 μl of unisp6, which equals 2 × 10^8^ copies, gave a Ct value about 28 cycles for unisp6 amplification. Since Ct values of over 35 cycles are considered as undetectable level, a minimum of ~10^6^ copies of unisp6 in plasma will be having a maximum Ct of 35 cycles by calculation. As such, 10^6^ copies of miRNA can be considered as sensitivity limit for the method. In order to confirm this sensitivity limit, additional experiments were performed by adding 0 μl, 0.25 μl, 1.0 μl of exogenous unisp6 into same volume of plasma, followed by a direct quantification of unnisp6 and 5s rRNA. As shown in Supplementary Figure S7, Ct values of 30 cycles and 28 cycles corresponded to 0.25 μl and 1.0 μl of unisp6, respectively. It is consistent with other data demonstrating the sensitivity limit of 10^6^ copies of miRNAs.

### The efficiency of the plasma-based direct quantification of circulating miRNAs

**Figure 4:**
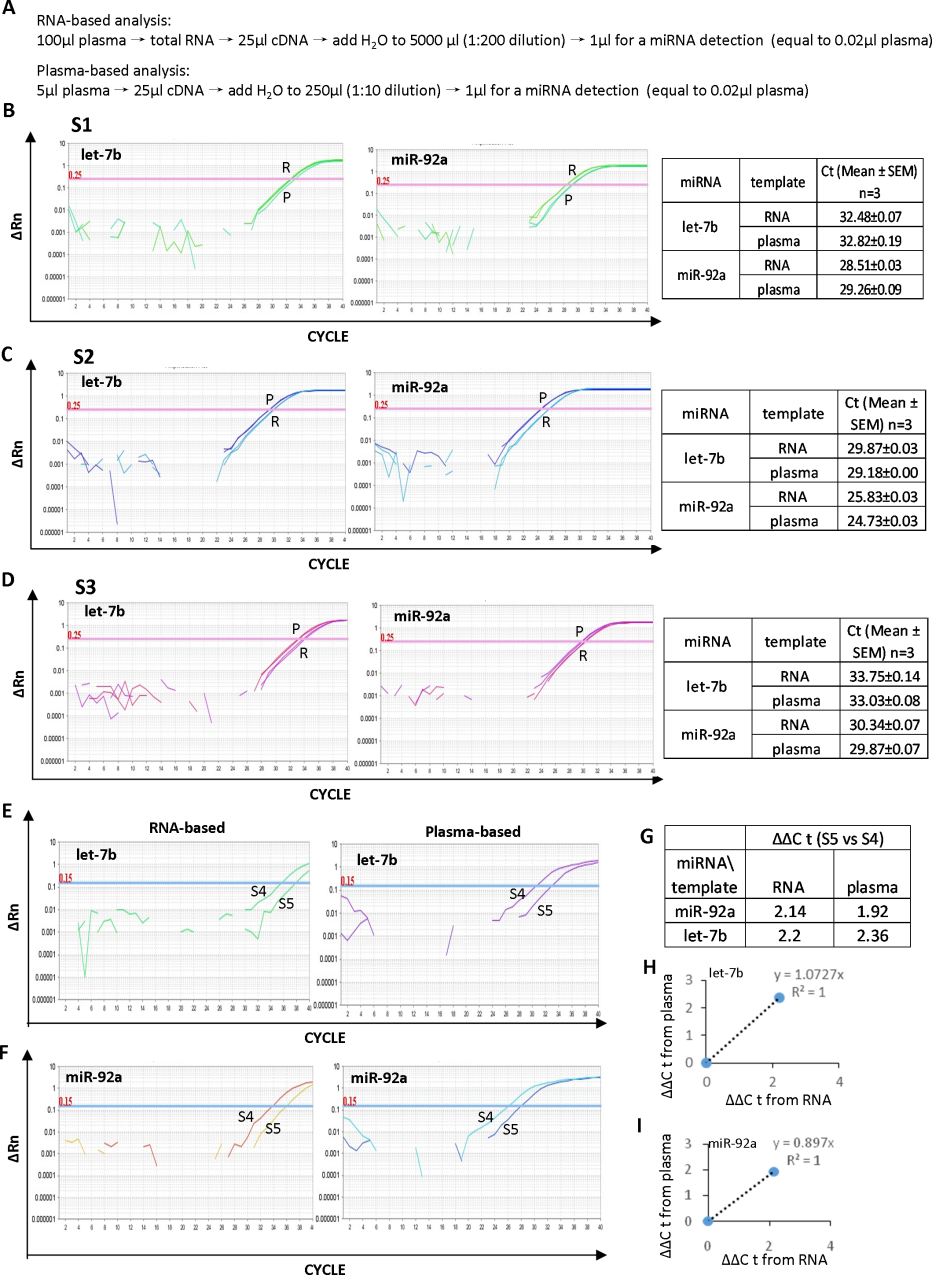
High efficiency of the plasma-based direct quantification of circulating miRNAs (**A**) Details of the procedure for the RNA-based and plasma-based miRNA analysis for comparison of the two methods. (**B**–**D**) Comparison of the RNA-based and plasma-based analysis for circulating let-7b and miR-92a in three independent samples S1 (B), S2 (C) and S3 (D). E-F: Comparison of the RNA-based and plasma-based analysis for the different expression of let-7b (**E**) and miR-92a (**F**) in plasma samples S4 and S5. (**G**) ΔΔCt value of let-7b and miR-92abetween samples S4 and S5. H-I: The linear correlation with R^2^ equals 1.0 between the RNA-based and plasma-based methods for the different expression analysis of let-7b (**H**) and miR-92a (**I**) in samples S4 and S5.

In order to determine the efficiency of the plasma-based method for measuring circulating miRNAs, selected plasma samples were either treated with Trizol to isolate RNA or treated following the M3 protocol to release miRNA from protein complexes. The RNA-based and plasma-based methods were compared for circulating miRNA analysis in three randomly selected plasma samples as shown in Figure 4A–4D. For each sample, identical amounts of cDNA either derived from purified RNA or derived directly from plasma were used to measure let-7b and miR-92a, two circulating miRNAs related to cancer. In both sample S2 and S3, the plasma-based direct quantification method showed smaller Ct values, ~0.7–1.1 cycles of ΔCt compared to the RNA-based method (Figure 4C and 4D). It is indicative of a higher efficiency of the plasma-based direct quantification method for circulating miRNA analysis.

In addition, the expression of circulating let-7b and miR-92a was analyzed in two randomly selected plasma samples (S4 and S5) and compared using either the RNA-based method or the plasma-based direct quantification method (Figure 4E and 4F). Both methods indicated higher levels of let-7b and miR-92a in S4 than that in S5 (Figure 4G). Furthermore, for both let-7b and miR-92a, ΔΔCt value between the two methods showed a linear correlation with R^2^ equals 1.0 (Figure 4H and 4I), demonstrating the high accuracy of the plasma-based direct quantification of circulating miRNA.

The stability of circulating miRNAs and the reproducibility of the plasma-based direct quantification method were also tested using plasma samples stored at a −80°C stored at day 1 and day 5 to measure the abundance of miR-16 (Supplementary Figure S8) and let-7b (Supplementary Figure S9). The Ct value showed strong stability for both miRNA at day 1 and day 5, indicating the stability of circulating miRNAs and the reproducibility of the direct quantitation method for miRNA analysis.

### Identification of circulating miRNAs as novel biomarkers for detecting metastatic breast cancer

**Figure 5:**
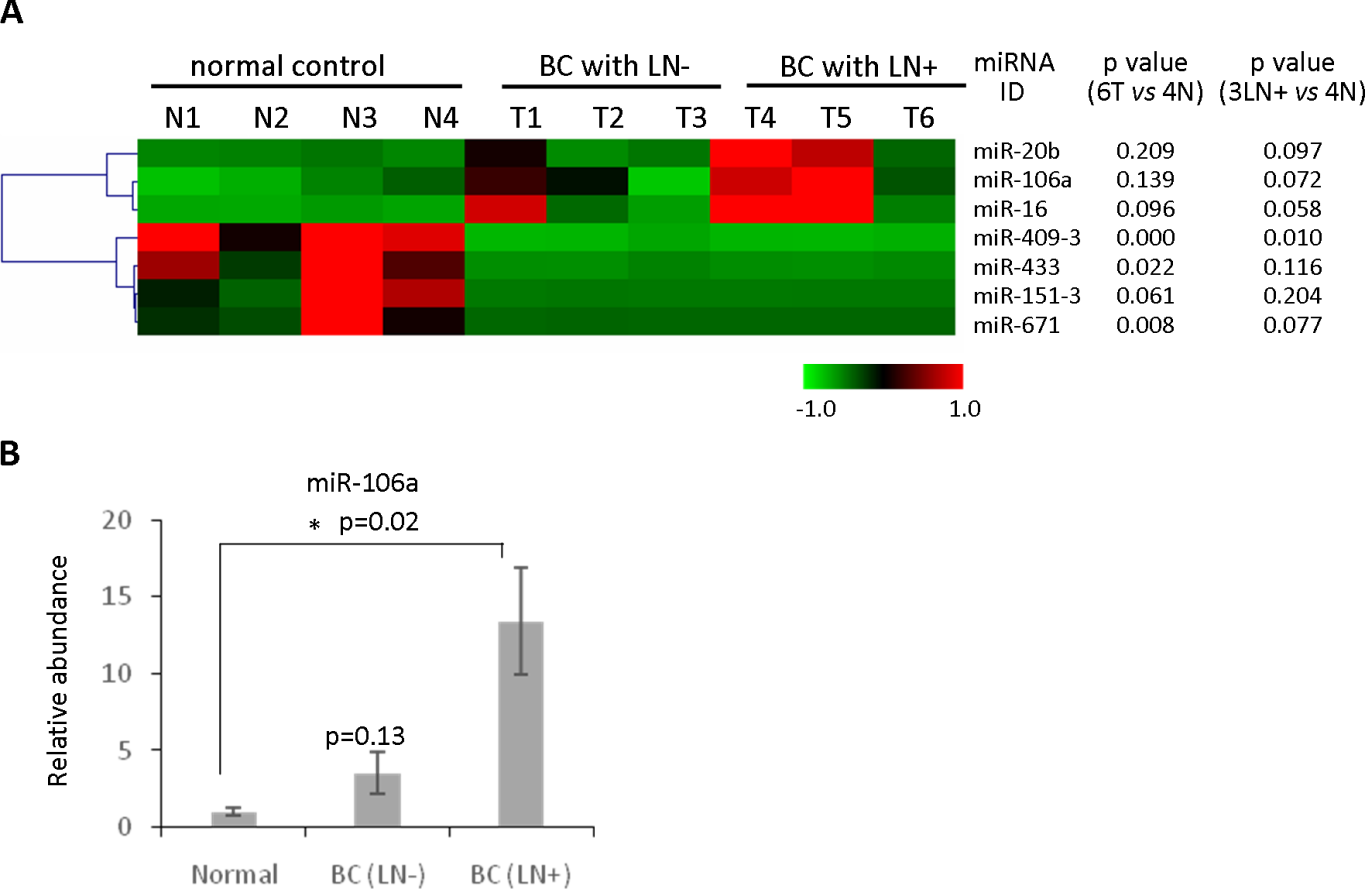
Identification of circulating miRNAs as novel biomarkers for detecting metastatic breast cancer (**A**) Heatmap of the circulating miRNA profiling analysis using purified RNAs from three lymph node positive (LN+) and three lymph node negative (LN−), as well as four normal control from age-matched healthy females. Three significantly upregulated circulating miRNAs (miR-20b, miR-106aand miR-16) and four downregulated circulating miRNAs (miR-671, miR-151-3p, miR-433 and miR-409-3p) in breast cancer were identified. (**B**) The method of plasma-based direct quantification of circulating miRNAs was applied to validate the miRNA signature in A, demonstrating significant upregulation of miR-106a in lymph node positive breast cancer patients compared with normal control.

Emerging evidence has demonstrated the potential of circulating miRNAs as biomarkers for early detection of cancer. We applied the plasma-based direct quantification method combined with the RNA-based method to validate a circulating miRNA signature in metastatic breast cancer. Total RNA was isolated from six plasma specimens of breast cancer patients including three lymph node positive (LN+) and three lymph node negative (LN−). Comparison was made with four normal control samples from age-matched healthy females. QRT-PCR based miRNA detection panels, which contain 365 cancer related miRNAs, were interrogated to detect the circulating miRNA profile in the six cancer specimens and the four normal controls. The results found three significantly upregulated circulating miRNAs (miR-20b, miR-106a and miR-16) and four downregulated circulating miRNAs (miR-671, miR-151-3p, miR-433 and miR-409-3p) in breast cancer (Figure 5A). In order to further validate the results, the plasma-based direct quantification method was applied to measure the abundance of these miRNAs in an additional three LN+, three LN− and three normal control plasma samples. The upregulation of miR-106a was confirmed in plasma samples from metastatic breast cancer patients (Figure 5B), which is consistent with the circulating miRNA screening results (Figure 5A). Notably, upregulation trend of circulating miR-20b (Supplementary Figure S10) and downregulation trend of circulating miR-671 were observed in the metastatic breast cancer patients. Circulating miR-409-3p showed downregulation trend in both LN+ and LN− breast cancer patients (Supplementary Figure S11). However *p* value did not show significance change due to the small number of samples. Still these results provided a subset of candidate circulating miRNAs as potential biomarkers for metastatic breast cancer.

In order to further confirm the upregulation of circulating miR-106a in metastatic breast cancer patients, additional plasma samples from 10 metastatic breast cancer patients and 10 control females were collected to test and compare the levels of circulating miR-106a. As shown in Supplementary Figure S12, the upregulation of circulating miR-106a in metastatic breast cancer patients was confirmed. Although the upregulation of circulating miR-20b and downregulation of Circulating miR-671 showed the trend in the metastatic breast cancer, it still needs further validation by analysis on an enlarged set of samples.

## DISCUSSION

Although nucleic acids was first demonstrated in human blood in 1948 [27], the source, structure and function of the nucleic acids and their relationship to human diseases was not exploited until early this century [28, 29]. The nucleic acids in plasma and serum include DNA, mRNA, miRNA and lncRNA. The nucleic acids may be released from blood cells, necrosed cell and tissues, apoptotic cells, exosomes and DNA/RNA-lipoprotein complexes [30]. Changes in the levels of circulating nucleic acids have been associated with pathological conditions including cancer progression. Circulating nucleic acids have been demonstrated to have potential roles in personalized disease diagnosis, prognosis and monitoring of therapeutic efficacy. In addition, nucleic acids in blood may also enter cells and exhibit a biological activity in the recipient cells [17].

Most of the circulating nucleic acids are protected from ribonuclease degradation by association with proteins, lipids or lipoprotein complexes, such as microvesicles and/or exosomes. Microvesicles, also named microparticles, are derived from plasma membranes with size ranging from 100 nm to 1000 nm. Exosomes are cell-derived circulating vesicles carrying cell-secreted nucleic acids and proteins. Exosomes have a smaller diameter of between 30 and 100 nm. Circulating miRNAs are protected by protein complexes in forms of microvesicles or exosomes. Releasing miRNAs from these particles with high efficiency is essential to quantify circulating miRNAs. Several methods have been successfully applied to quantify circulating miRNAs including miRNA array, miRNA real-time PCR, and high-throughput small RNA-SEQ. These methods are based on RNA isolation or small RNA enrichment from serum or plasma. Isolation of small miRNA molecules is inefficient when using the regular RNA isolation methods such as RNA purification columns. Although Trizol reagent is formulated for mRNA isolation, it is applicable for miRNA extraction. However, it is unavoidable to have some of the circulating miRNAs lost due to incomplete protein denaturation and/or incomplete RNA precipitation because of the high concentration of proteins serum and plasma. Therefore, a quantitative analysis of circulating miRNAs with low abundances becomes inefficient.

The feasibility of quantifying the gene expression level directly from cell lysates (cell-to-Ct) was primarily tested and reported in 2013 [25], where multiple reagents were tested and compared for quantification analysis of both mRNA and miRNA. Asaga and Hoon reported a protocol for direct quantifying circulating miRNA from serum [26] but lacking the accuracy and specificity examination. Herein we applied a direct quantification method combining chemical denaturation and heat denaturation to release circulating miRNAs from both microvesicles and exosomes followed by a direct quantitative PCR amplification. We show this method is more efficient with higher accuracy and specificity for circulating miRNA analysis, compared with the traditional purified RNA-based RT-PCR method.

Circulating miRNAs have potential as biomarkers for cancer diagnosis and prognosis. Developing a convenient and reliable method with high accuracy and specificity to measure circulating miRNAs will be of great benefit to both laboratory screening and clinical applications. The plasma-based method was successfully applied herein to breast cancer clinical samples for screening and validating of circulating miRNAs as biomarkers for metastatic breast cancer. Changes of miR-106a levels in breast cancer patients' blood were correlated with metastatic status. Although miRNAs in blood as potential biomarkers for breast cancer has been frequently reported [23, 24], it is rare for validation of circulating miRNA as a biomarker for metastatic cancer. In addition to miR-106a, a subset of circulating miRNAs including miR-20b and miR-671, were screened and primarily tested as biomarker candidates for metastatic breast cancer. More clinical samples are required to further validate the predictive value of these candidates for metastatic breast cancer before clinical application.

## MATERIALS AND METHODS

### Plasma collection

Blood samples were collected from breast cancer patients and age-matched healthy females at the Shanghai East hospital, Department of Surgery. 5 ml of blood were collected in EDTA-treated blood-collecting tubes followed by a centrifugation at speed of 2,000 rpm for 5 min at 4°C. The supernatant was aliquoted and stored at −80°C. All the procedures were approved by the Institutional Review Board (IRB) of Shanghai East Hospital, Tongji University School of Medicine.

### Plasma preparation for miRNA measurement

For circulating miRNA analysis, 5 μl of plasma was mixed with 5 μl of denaturing buffer composed of 2.5% tween-20, 50 mM Tris and 1 mM EDTA, followed by heating in a water bath at 75°C for 5 min, then cooling on ice, and centrifuging at a speed of 12,000 rpm for 10 min at 4°C. The supernatant was used for miRNA reverse transcription and real-time PCR.

### miRNA QRT-PCR analysis

A M & G miRNA Reverse Transcription kit (miRGenes, Shanghai, China) was used to prepare the first strand cDNA of plasma miRNAs following the manufacturer's instruction. 5 μl of prepared plasma solution or 100 ng of purified total RNA from each plasma specimen was used for miRNA measurement. After reverse transcription, the cDNA was diluted 1:10-100 (for plasma method) or 1:200-1000 (for purified RNA method) for real-time PCR. The sequences of forward primers for miR-92a, miR-16, miR-106a, miR-671, let-7b and 5s rRNA are: miR-92a: attgcacttgtcccggcctg; miR-16: agcagcacgtaaatattggc; miR-106a: agtgcttacagtgcaggtag; miR-671: aggaagcccuggagg; let-7b: tgaggtagtaggttgtgtg; 5s rRNA: agtacttggatgggagaccg. The DNA oligoes were synthesized and purified by GenScript (Nanjing, China). A universal reverse primer was provided by miRGenes. The spike-in RNA unisp6 and PCR primers for amplify unisp6 were purchased from Exiqon. 1 μl of unisp6 equals 10^8^ copies. The SYBR Green Master Mix was ABI product (Applied Biosystem, Life Technologies). The ABI 7900 HT and ABI 7500 Sequence Detection System (Applied Biosystem, Life Technologies) were used for quantitative real time PCR assay. 5s rRNA was used for normalization when necessary.

### miRNA profiling analysis in plasma of breast cancer patients and normal control

We customized a human cancer-related miRNA panel which contains 365 human cancer-related miRNAs and 5 reference small RNAs, which was prepared by miRGenes (Shanghai, China). Total RNA was isolated using Trizol reagent (Life Technologies) from plasma specimens. The cDNA was prepared as described above. The miRNA expression profiles in plasma specimens were performed using the human cancer-related miRNA panel, based on the quantitative real-time PCR methods. The result was analyzed by Mev (version 4.9) software. *p* < 0.10 was considered significant.

### Statistical analysis

Data are presented as mean ± SEM. The standard two-tailed Student's *t*-test was used for statistical analysis, in which *P* < 0.05 was considered significant.

## SUPPLEMENTARY MATERIALS FIGURES



## References

[1] International Human Genome Sequencing Consortium (2004). Finishing the euchromatic sequence of the human genome. Nature.

[2] Cui Q, Yu Z, Purisima EO, Wang E (2006). Principles of microRNA regulation of a human cellular signaling network. Mol Syst Biol.

[3] Tili E, Michaille JJ, Gandhi V, Plunkett W, Sampath D, Calin GA (2007). miRNAs and their potential for use against cancer and other diseases. Future Oncol.

[4] Calin GA, Croce CM (2006). MicroRNA signatures in human cancers. Nat Rev Cancer.

[5] Calin GA, Sevignani C, Dumitru CD, Hyslop T, Noch E, Yendamuri S, Shimizu M, Rattan S, Bullrich F, Negrini M, Croce CM (2004). Human microRNA genes are frequently located at fragile sites and genomic regions involved in cancers. Proceedings of the National Academy of Sciences of the United States of America.

[6] Zhang B, Pan X, Cobb G.P, Anderson TA (2007). microRNAs as oncogenes and tumor suppressors. Dev Biol.

[7] Esquela-Kerscher A, Slack FJ (2006). Oncomirs - microRNAs with a role in cancer. Nat Rev Cancer.

[8] Cummins JM, Velculescu VE (2006). Implications of micro-RNA profiling for cancer diagnosis. Oncogene.

[9] Kosaka N, Izumi H, Sekine K, Ochiya T (2010). microRNA as a new immune-regulatory agent in breast milk. Silence.

[10] Lawrie CH, Gal S, Dunlop HM, Pushkaran B, Liggins AP, Pulford K, Banham AH, Pezzella F, Boultwood J, Wainscoat JS, Hatton CS, Harris AL (2008). Detection of elevated levels of tumour-associated microRNAs in serum of patients with diffuse large B-cell lymphoma. Br J Haematol.

[11] Mitchell PS, Parkin RK, Kroh EM, Fritz BR, Wyman SK, Pogosova-Agadjanyan EL, Peterson A, Noteboom J, O'Briant KC, Allen A, Lin DW, Urban N, Drescher CW (2008). Circulating microRNAs as stable blood-based markers for cancer detection. Proceedings of the National Academy of Sciences of the United States of America.

[12] Chen X, Ba Y, Ma L, Cai X, Yin Y, Wang K, Guo J, Zhang Y, Chen J, Guo X, Li Q, Li X, Wang W (2008). Characterization of microRNAs in serum: a novel class of biomarkers for diagnosis of cancer and other diseases. Cell Res.

[13] Arroyo JD, Chevillet JR, Kroh EM, Ruf IK, Pritchard CC, Gibson DF, Mitchell PS, Bennett CF, Pogosova-Agadjanyan EL, Stirewalt DL, Tait JF, Tewari M (2011). Argonaute2 complexes carry a population of circulating microRNAs independent of vesicles in human plasma. Proceedings of the National Academy of Sciences of the United States of America.

[14] Katsuda T, Kosaka N, Ochiya T (2014). The roles of extracellular vesicles in cancer biology: toward the development of novel cancer biomarkers. Proteomics.

[15] Kosaka N, Iguchi H, Ochiya T (2010). Circulating microRNA in body fluid: a new potential biomarker for cancer diagnosis and prognosis. Cancer Sci.

[16] Yu Z, Willmarth NE, Zhou J, Katiyar S, Wang M, Liu Y, McCue PA, Quong AA, Lisanti MP, Pestell RG (2010). microRNA 17/20 inhibits cellular invasion and tumor metastasis in breast cancer by heterotypic signaling. Proceedings of the National Academy of Sciences of the United States of America.

[17] Turchinovich A, Samatov TR, Tonevitsky AG, Burwinkel B (2013). Circulating miRNAs: cell-cell communication function?. Front Genet.

[18] Chang EL, Lo S (2003). Diagnosis and management of central nervous system metastases from breast cancer. The oncologist.

[19] Sanchez-Munoz A, Perez-Ruiz E, Ribelles N, Marquez A, Alba E (2008). Maintenance treatment in metastatic breast cancer. Expert review of anticancer therapy.

[20] Yu Z, Wang C, Wang M, Li Z, Casimiro MC, Liu M, Wu K, Whittle J, Ju X, Hyslop T, McCue P, Pestell RG (2008). A cyclin D1/microRNA 17/20 regulatory feedback loop in control of breast cancer cell proliferation. The Journal of cell biology.

[21] Iorio MV, Ferracin M, Liu CG, Veronese A, Spizzo R, Sabbioni S, Magri E, Pedriali M, Fabbri M, Campiglio M, Ménard S, Palazzo JP, Rosenberg A (2005). MicroRNA gene expression deregulation in human breast cancer. Cancer research.

[22] Zhang L, Huang J, Yang N, Greshock J, Megraw MS, Giannakakis A, Liang S, Naylor TL, Barchetti A, Ward MR, Yao G, Medina A, O'brien-Jenkins A (2006). microRNAs exhibit high frequency genomic alterations in human cancer. Proceedings of the National Academy of Sciences of the United States of America.

[23] Heneghan HM, Miller N, Lowery AJ, Sweeney KJ, Newell J, Kerin MJ (2010). Circulating microRNAs as novel minimally invasive biomarkers for breast cancer. Ann Surg.

[24] Ferracin M, Lupini L, Salamon I, Saccenti E, Zanzi MV, Rocchi A, Da Ros L, Zagatti B, Musa G, Bassi C, Mangolini A, Cavallesco G, Frassoldati A (2015). Absolute quantification of cell-free microRNAs in cancer patients. Oncotarget.

[25] Ho Y.K, Xu WT, Too HP (2013). Direct quantification of mRNA and miRNA from cell lysates using reverse transcription real time PCR: a multidimensional analysis of the performance of reagents and workflows. PloS one.

[26] Asaga S, Hoon DS (2013). Direct serum assay for microRNA in cancer patients. Methods Mol Biol.

[27] Mandel P, Metais P (1948). Les acids nucleiques du plasma sanguine chez l'homme. C R Seances Soc Biol Fil.

[28] Rainer TH, Wong LK, Lam W, Yuen E, Lam NY, Metreweli C, Lo YM (2003). Prognostic use of circulating plasma nucleic acid concentrations in patients with acute stroke. Clin Chem.

[29] Lam NY, Rainer TH, Chan LY, Joynt G.M, Lo YM (2003). Time course of early and late changes in plasma DNA in trauma patients. Clin Chem.

[30] Gahan PB, Stroun M, Rykova EY, Kikuchi Y (2010). The biology of circulating nucleic acids in plasma and serum. Extracellular nucleic acids.

