# Supplementary material for: A direct quantification method for measuring plasma MicroRNAs identified potential biomarkers for detecting metastatic breast cancer
Source: Oncotarget. 2016 Mar 8;7(16):21865–74. doi: 10.18632/oncotarget.7990 (PMC5008329; doi:10.18632/oncotarget.7990)
Supplement: Supplementary file 1 [file oncotarget-07-21865-s001.pdf]

A direct quantification method for measuring plasma MicroRNAs identified potential biomarkers for detecting metastatic breast cancer

Supplementary Materials

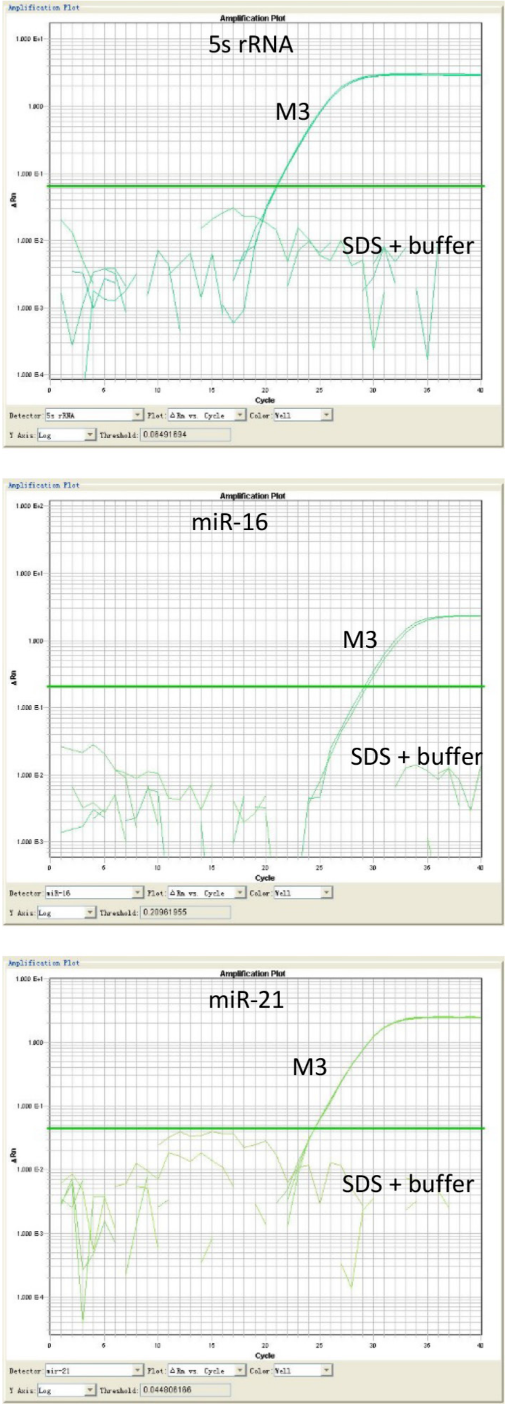

Supplementary Figure S1: Quantitative analysis of 5s rRNA, miR-16 and miR-21 in plasma sample treated with 10% SDS or method M3.

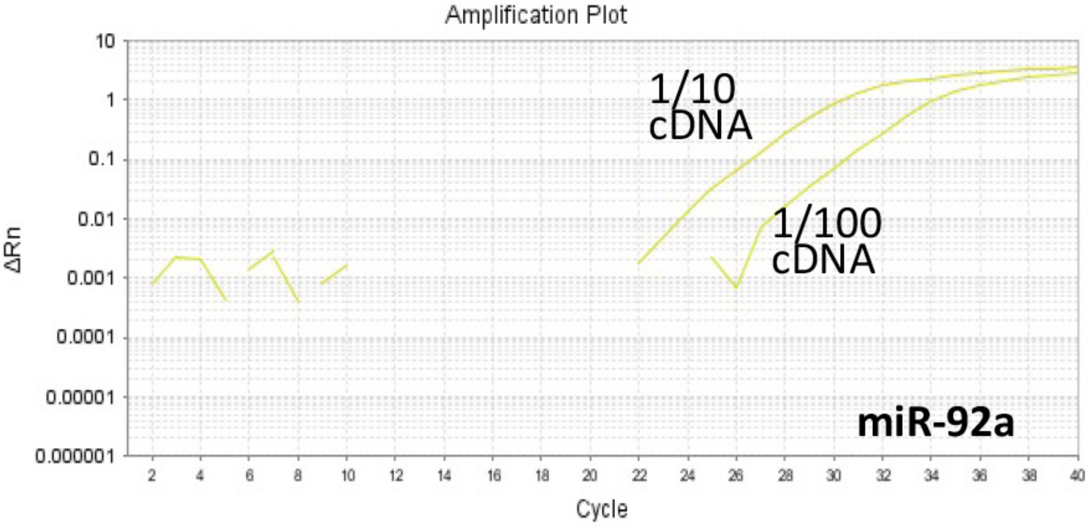

Supplementary Figure S2: Quantitative analysis of miR-92a in plasma sample with 1:10 and 1:100 cDNA dilutions using the method M3.

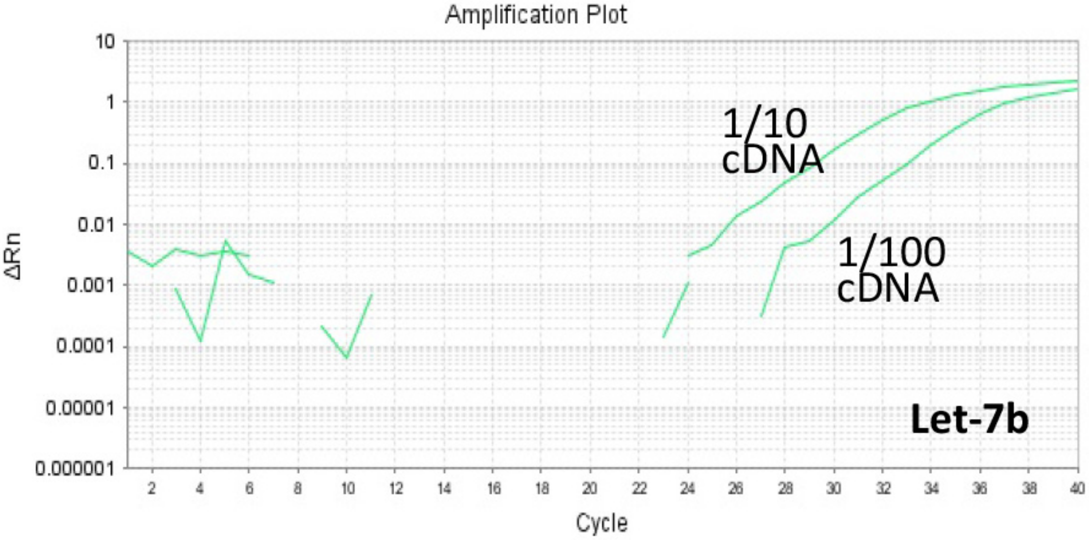

Supplementary Figure S3: Quantitative analysis of let-7b in plasma sample with 1:10 and 1:100 cDNA dilutions using the method M3.

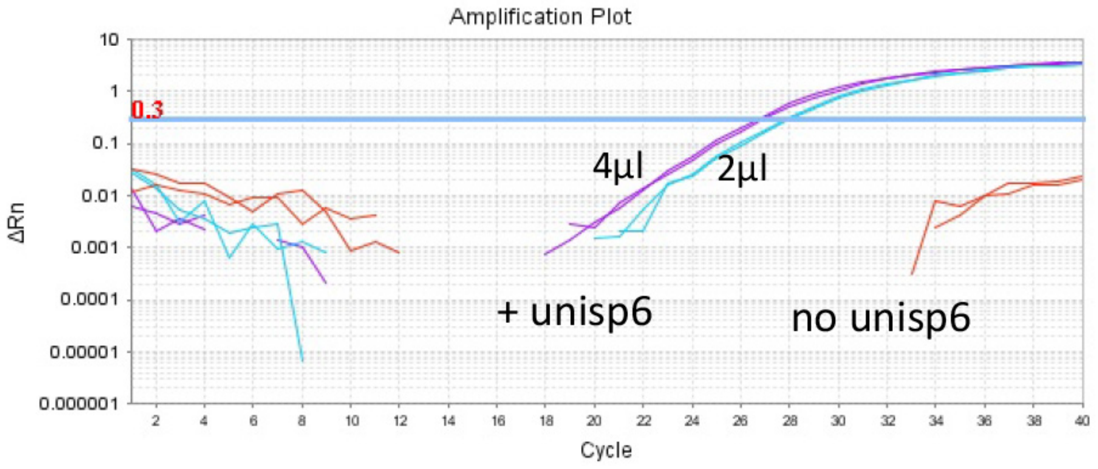

Supplementary Figure S4: Quantitative analysis of exogenous small RNA unisp6 which was added into plasma with volume 0, 2 and 4 µl.

unisp6 (spike-in)	Ct_{sp6}
0	Undetermined
2 µl	27.83 ± 0.13
4 µl	26.69 ± 0.15

Supplementary Figure S5: Ct values for unisp6 amplification in S4.

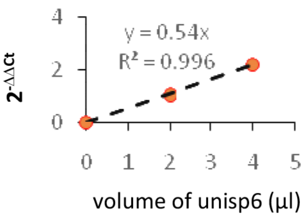

Supplementary Figure S6: Linear positive correlation in S4 between the relative abundance of unisp6 and the unisp6 concentration in plasma with R2 = 0.997.

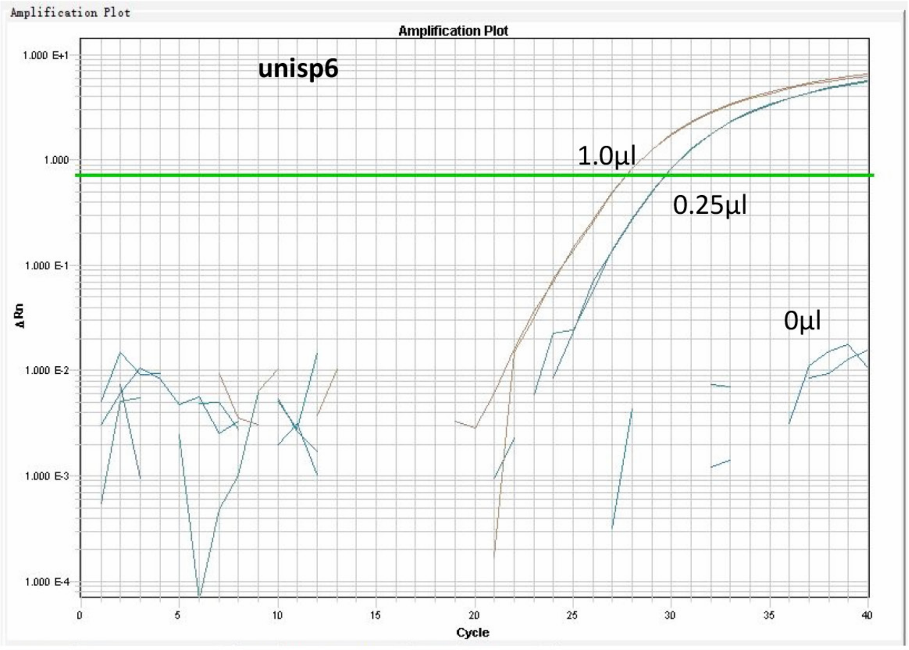

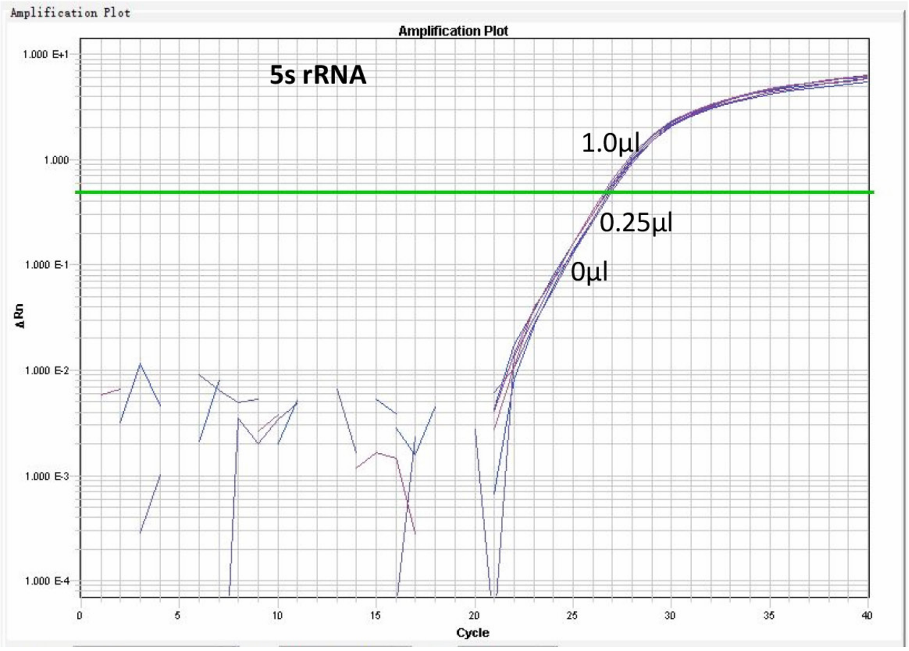

Supplementary Figure S7: Quantitative analysis of exogenous small RNA unisp6 which was added into plasma with volume 0, 0.25 and 1 µl. 5s rRNA was analyzed as well.

Date	ΔCt (miR-16 vs 5s rRNA) (mean ± SEM)
day 1	4.15 ± 0.04
day 5	4.12 ± 0.23

Supplementary Figure S8: Ct values for miR-16 amplification in a plasma sample at day 1 and day 5 using the method M3.

Date	ΔCt (let-7b vs 5s rRNA) (mean ± SEM)
day 1	4.53 ± 0.41
day 5	4.30 ± 0.06

Supplementary Figure S9: Ct values for let-7b amplification in a plasma sample at day 1 and day 5 using the method M3.

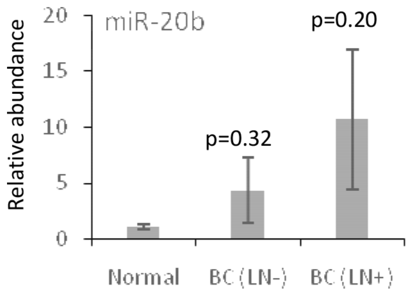

Supplementary Figure S10: The relative abundance of miR-20b in the plasma samples from three LN+ breast cancer patients, three LN− breast cancer patients and three normal controls.

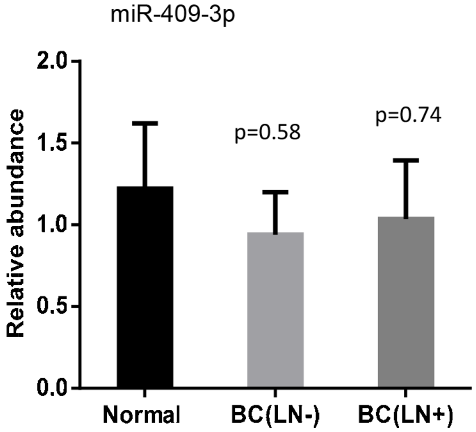

Supplementary Figure S11: The relative abundance of miR-409-3p in the plasma samples from three LN+ breast cancer patients, three LN− breast cancer patients and three normal controls.

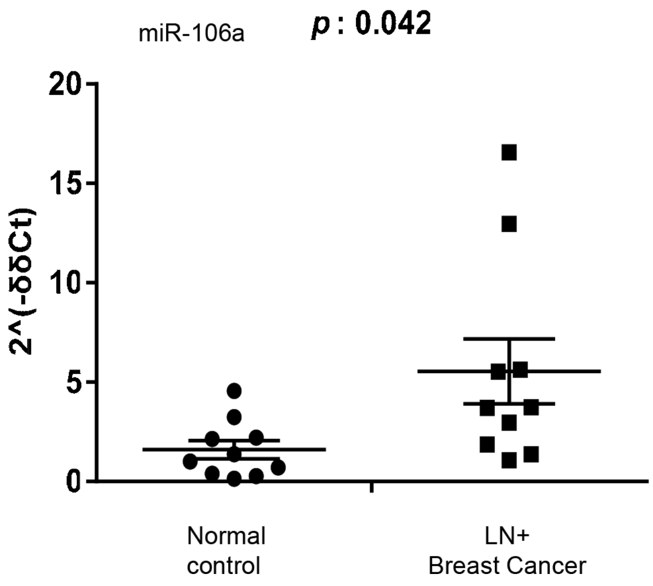

Supplementary Figure S12: The relative abundance of miR-106a in the plasma samples from additional 10 LN+ breast cancer patients and 10 normal controls.
